# Functional roles of SRC signaling in pancreatic cancer: Recent insights provide novel therapeutic opportunities

**DOI:** 10.1038/s41388-023-02701-x

**Published:** 2023-04-29

**Authors:** Ashleigh R. Poh, Matthias Ernst

**Affiliations:** Olivia Newton-John Cancer Research Institute and La Trobe University School of Cancer Medicine, Melbourne, VIC 3084 Australia

**Keywords:** Pancreatic cancer, Cancer microenvironment

## Abstract

Pancreatic ductal adenocarcinoma (PDAC) is an aggressive malignant disease with a 5-year survival rate of <10%. Aberrant activation or elevated expression of the tyrosine kinase c-SRC (SRC) is frequently observed in PDAC and is associated with a poor prognosis. Preclinical studies have revealed a multifaceted role for SRC activation in PDAC, including promoting chronic inflammation, tumor cell proliferation and survival, cancer cell stemness, desmoplasia, hypoxia, angiogenesis, invasion, metastasis, and drug resistance. Strategies to inhibit SRC signaling include suppressing its catalytic activity, inhibiting protein stability, or by interfering with signaling components of the SRC signaling pathway including suppressing protein interactions of SRC. In this review, we discuss the molecular and immunological mechanisms by which aberrant SRC activity promotes PDAC tumorigenesis. We also provide a comprehensive update of SRC inhibitors in the clinic, and discuss the clinical challenges associated with targeting SRC in pancreatic cancer.

## Pancreatic ductal adenocarcinoma (PDAC)

PDAC is an aggressive malignant disease that accounts for more than 90% of pancreatic cancer cases [1]. Due to nonspecific clinical symptoms, most patients are diagnosed at advanced stages of the disease, and are not eligible for surgery [2, 3]. Systemic chemotherapy is commonly employed as the first-line treatment in patients with nonresectable tumors; however, durable responses are observed in <30% of cases [4, 5]. Furthermore, immunotherapies have failed to translate into meaningful improvements in a majority of PDAC patients due to the presence of a highly immunosuppressive and desmoplastic tumor microenvironment [6–8]. Given the poor prognosis and limited treatment options for PDAC, a better understanding of key signaling pathways and molecules involved in tumor initiation, development, and metastasis is crucial to guide the use of existing therapies and identify new drug targets.

## SRC

Francis Peyton Rous was awarded the Nobel Prize in 1966 for his discovery of the transmissible avian Rous sarcoma virus (RSV), which provided the first evidence of virally-mediated tumorigenesis [9]. Subsequently, it was shown that the genome of RSV encoded a tyrosine kinase referred to as viral SRC (*v-Src)*, which stimulated uncontrolled proliferation in host cells [10, 11]. In 1979, J. Michael Bishop and Harold Varmus discovered that normal chicken cells possessed a cellular homolog of v-Src referred to as cellular SRC (*c-Src*) that shared striking resemblance to *v-Src* [10]. Importantly, RSV had integrated a genomic sequence encoding a truncated version of *c-Src* that lacked the regulatory carboxy-terminal tail. Thus, unlike its cellular counterpart c-SRC, which was referred to as a “proto-oncogene”, v-SRC remained constitutively active [12]. These insights led to a paradigm shift in oncology by demonstrating that mutations in tightly regulated proteins encoded by proto-oncogenes can also promote tumor development, with or without viral involvement.

### SRC structure and activation

c-SRC (SRC) belongs to a family of nine non-receptor tyrosine kinases collectively referred to as the SRC family kinase (SFK), which comprises three distinct groups based on the expression pattern of individual members. The first group (SRC, FYN, and YES) are ubiquitously expressed. The second group (HCK, BLK, FGR, LCK, YRK, and LYN) are primarily expressed in hematopoietic cells, whereas the third group (FRK) is predominantly expressed on epithelial cells [13].

**Fig. 1:**
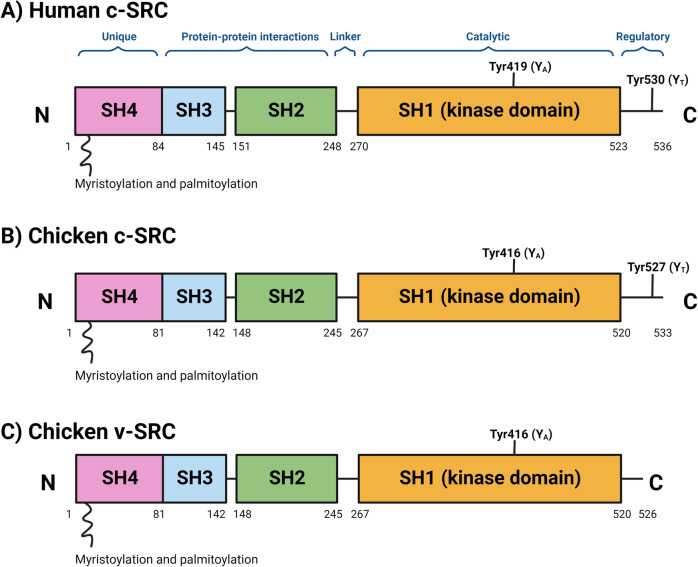
Structure of SRC. Schematic of (**A**) human c-SRC, (**B**) chicken c-SRC and **(C)** chicken v-SRC. Human c-SRC is comprised of a 14-carbon myristic acid moiety attached to the N-terminal SRC Homology 4 domain (SH4), a unique domain, followed by an SH3 and SH2 domain, a linker, a SH1 protein kinase domain containing a tyrosine residue at Tyr419 (Y_A_; Tyr416 in chickens), and a C-terminal negative regulatory tail containing a tyrosine residue at Tyr530 (Y_T_; Tyr527 in chickens). Chicken v-SRC lacks the C-terminal segment containing the Y_T_ residue, which renders it constitutively active and capable of inducing cellular transformation. *Figure created in Biorender*.

SFKs are comprised of a unique amino-terminal SRC Homology 4 domain (SH4), followed by conserved regulatory SH3 and SH2 domains, a flexible linker, a SH1 protein kinase domain containing a tyrosine residue in the activation loop (Y_A_), and a C-terminal tail containing a negative regulatory tyrosine residue (Y_T_) (Fig. 1) [14].

The SH4 domain contains myristoylation and palmitoylation sites that anchor SFKs to the inner leaf of the cytoplasmic membrane (Fig. 1). The SH3 domain is a small protein domain (61 amino acid residues in SRC), which is folded into β-barrels of five antiparallel strands. The SH3 domain mediates protein–protein interactions by binding to proline-rich motifs of client proteins [15], and is necessary for substrate recognition and to some extent regulation of kinase activity [16, 17]. Meanwhile, the SH2 domain (97 amino acid residues in SRC) is comprised of a β-sheet flanked by opposing α-helices [18]. The SH2 domain has a conserved arginine-containing recognition pocket that binds to short phosphotyrosine motifs [18]. Lastly, the SH1 catalytic domain contains the active site of the kinase domain nestled between a small N-lobe and a large C-lobe. The N-lobe consists of five-stranded antiparallel β-sheets and a regulatory αC-helix, while the C-lobe contains α-helical segments [19].

**Fig. 2:**
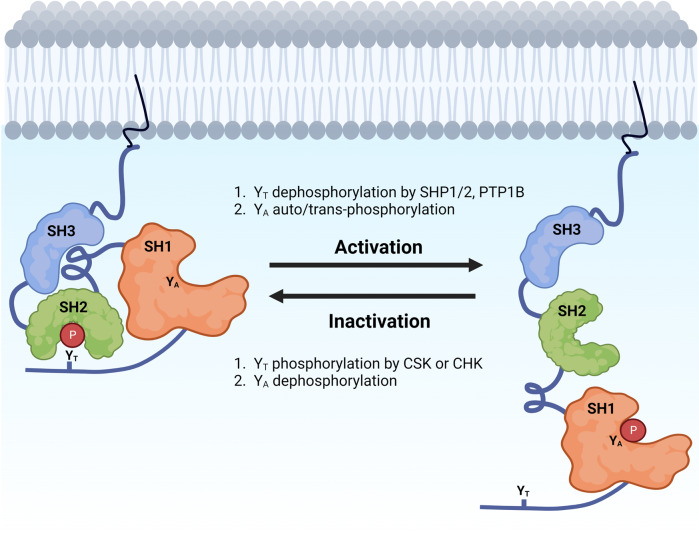
Regulation of SRC activation. SRC is maintained in an inactive conformation by the binding of the SH2 linker to the SH3 domain, and by the binding of the phosphorylated Y_T_ to the SH2 domain. Activation of SRC occurs following dephosphorylation of Y_T_, as well as auto/trans-phosphorylation of Y_A_. *Figure created in Biorender*.

SFKs exist in dynamic equilibria between active and inactive conformations. The inactive ‘closed’ conformation is maintained by two intra-molecular associations (Fig. 2). This closed conformation primarily results from the association of the SH2 domain to the phosphorylated Y_T_ residue, and is further strengthened by binding of the SH3 domain to the linker separating the SH2 and SH1 domains [20]. Phosphorylation of Y_T_ is tightly regulated by members of the C-terminal SRC kinase (CSK) family, including CSK and CHK [14, 20]. Since v-SRC lacks the C-terminal negative regulatory segment containing the Y_T_ residue, it is constitutively active and able to induce cellular transformation [14].

Activation of SFKs is mediated by dephosphorylation of Y_T_ and auto-phosphorylation of Y_A_ [21]. Dephosphorylation of Y_T_ by phosphatases such as protein tyrosine phosphatase (PTP)1, and SH2-containing phosphatases (SHP) 1 and SHP2 results in an ‘open’ conformation that facilitates the binding of the SH2 and SH3 domains with their corresponding ligands [14]. Subsequently, auto-phosphorylation of Y_A_ results in full SFK activation [22].

### Mechanisms underpinning aberrant SRC signaling in cancer

**Fig. 3:**
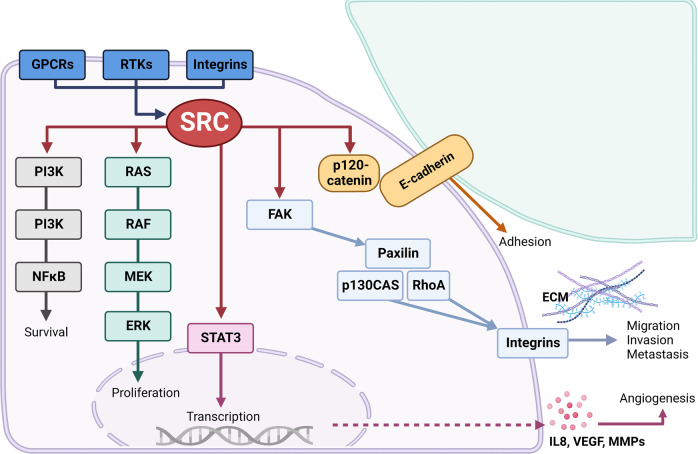
Examples of SRC signal transduction pathways. SRC can interact with G-protein coupled receptors (GPCRs), receptor tyrosine kinases (RTKs; e.g., MET, PDGFR, EGFR), and integrins to activate downstream signaling pathways (e.g., PI3K, RAS/ERK, STAT3) that promote cell proliferation, survival and angiogenesis. In addition, SRC can phosphorylate p120-catenin to disrupt adherens junctions stabilized by E-cadherin to enhance cell adhesion. Conversely, SRC and FAK signaling mediates the activation of downstream targets (e.g., p130CAS, Paxillin, RhoA) and results in the formation of complexes with integrin molecules that modulate the extracellular matrix (ECM) to stimulate cell migration, invasion, and metastasis. *Figure created in Biorender*.

SRC mediates a wide range of signaling pathways that play a pivotal role in normal cellular processes, including proliferation, adhesion, angiogenesis, and migration (Fig. 3). Given the contribution of these features to some of the “hallmarks of cancer,” it is unsurprising that aberrant activation or expression of SRC is observed in many tumor types and correlates with poor patient outcomes. However, while activating mutations in SRC are observed in <2% of pancreatic cancer patients [23–25], elevated expression, and/or activation of SRC in PDAC confers a weakly oncogenic outcome [26–28].

Aberrant activation of SRC can result from several mechanisms, including amplification of upstream signaling pathways, loss of negative regulation, and impaired degradation. Indeed, SRC directly interacts with a variety of cellular factors, including signal transducers and activators of transcription (STAT) proteins, cyclins, and tyrosine kinases (e.g., colony-stimulating factor 1 receptor (CSF1R), platelet derived growth factor receptor (PDGFR), epidermal growth factor receptor (EGFR), human epidermal growth factor receptor (HER), and focal adhesion kinase (FAK)) [29]. FAK signaling enables the fibrotic and immune suppressive tumor microenvironment of PDAC, and its elevated expression is associated with poor overall survival [30, 31]. Following integrin engagement or ligand stimulation, FAK forms a complex with SRC and triggers the activation of downstream pathways involved in tumor cell migration, invasion, survival, and immune suppression [27, 30, 32, 33]. Moreover, amplification of genes associated with SRC effector networks such as PI3K/AKT/mTor and FAK are reported in 17% and 6% of pancreatic tumors, respectively [23–25], while aberrant expression of integrin signaling components that activate SRC are observed in 67% of pancreatic tumors [34]. Collectively, these results highlight a mechanism by which cooperation of SRC with its binding partners may promote feed-forward loops to reinforce its kinase activity.

Aberrant SRC activity may also arise due to disruption to proteins that regulate its function. Gain-of-function mutations in SHP2 leads to increased SRC activation and tumor development in preclinical models [35–37], while reduced expression of CSK correlates with enhanced SRC activity in cancer cells [38]. Another possible mode of increased SRC activation includes altered protein stability, which may occur due to deregulation of the ubiquitin ligase c-CBL that mediates proteasomal degradation of SRC [39, 40].

## Effects of tumor cell-intrinsic SRC activity in pancreatic cancer

Tumor cell-intrinsic mechanisms by which SRC signaling facilitates PDAC development and progression include enhancing cancer cell growth and survival, promoting stemness, induction of metabolic reprogramming, facilitating tumor invasion and metastasis, and mediating chemoresistance (Fig. 3).

### Cancer cell growth and survival

#### RAS/RAF/MEK/ERK signaling

Activating mutations in KRAS are a hallmark of pancreatic cancer, and are observed in approximately 95% of patients [41, 42]. Under steady-state conditions, SRC phosphorylates RAS, and triggers downstream activation of the MAPK signaling cascade to stimulate cellular proliferation, differentiation, and survival [43]. Notably, pancreas-specific oncogenic Kras^G12D^ expression and deletion of the SRC negative regulator CSK accelerates PDAC development in mice compared to littermates that express Kras^G12D^ and wild-type levels of SRC [44].

#### PI3K signaling

SRC regulates PI3K signaling by directly phosphorylating the p85 subunit of PI3K [45], and by inhibiting the PI3K negative regulatory phosphatase and tensin homolog (PTEN) [46]. This results in downstream phosphorylation of AKT, which enhances the growth and survival of pancreatic cancer cells [47].

#### STAT3 signaling

Reciprocal activation of SRC and STAT3 is implicated in PDAC progression [48–50]. v-SRC directly associates with STAT3 to promote its phosphorylation, DNA binding, and transcriptional activity [51, 52]. Reciprocally, STAT3 activation is required for v-SRC to promote cellular transformation [53–55]. SRC and STAT3 signaling enhances the transcription of angiogenic genes (e.g., *IL8*, *VEGF*) in human pancreatic cancer cells [56–58], while inhibition of SRC activity decreases STAT3 phosphorylation and tumorigenicity [56].

#### YAP signaling

YES1-associated protein (YAP) is a major effector of the Hippo signaling pathway, and enables an immune suppressive PDAC microenvironment [59, 60]. SRC plays a major role for YAP nuclear localization and phosphorylation [61], which results in the transcription of YAP-target genes involved in cell proliferation and survival through Hippo-dependent [62, 63] and Hippo-independent pathways [64].

#### FAK signaling

Aberrant FAK activation facilitates cancer cell migration, survival, adhesion, and invasion, and is associated with a worse prognosis in PDAC [65–67]. Following integrin engagement or ligand stimulation, FAK undergoes auto-phosphorylation and forms a complex with SRC [68, 69], which induces a conformational change in SRC and its activation. In turn, activated SRC phosphorylates FAK, which serves as a docking site for proteins that stimulate the activation of downstream signaling pathways including RAS, STAT3, and PI3K [70]. The FAK and SRC complex also phosphorylates cytoskeletal adapter proteins (e.g., Paxillin and CAS), which recruit and activate additional signaling molecules involved in cell motility and invasion [69].

#### Crosstalk with receptor tyrosine kinases (e.g., EGFR and PDGFR)

Aberrant activation of SRC and EGFR is observed in most human malignancies, suggesting functional cooperativity to promote tumor development [71]. In addition to direct tyrosine phosphorylation [72–74], SRC can indirectly activate EGFR signaling by stimulating matrix metalloproteinases (MMPs), which cleave EGFR ligands to promote receptor activation [75]. Another mechanism by which SRC amplifies EGFR signaling is by promoting destruction of the ubiquitin-protein ligase c-CBL, which mediates proteasomal degradation of activated EGFR [76, 77].

In support of the oncogenic synergy between SRC and EGFR in PDAC, stable complexes between SRC and EGFR contribute to more aggressive tumor phenotypes by enhancing DNA synthesis and mitosis [71, 78]. Accordingly, dasatinib (SRC inhibitor) and erlotinib (EGFR inhibitor) treatment reduces pancreatic cancer cell migration and invasion, overcomes STAT3-mediated chemoresistance, and attenuates the growth of PDAC xenografts [79].

SRC also plays versatile roles in mediating cell responses regulated by PDGFR signaling, including cell survival, migration, and actin cytoskeleton rearrangement [80, 81]. Ligand-induced activation of PDGFRβ results in dephosphorylation of SRC on Tyr530 and auto-phosphorylation on Tyr419. In turn, SRC phosphorylates PDGFR and renders it active [82–84]. Constitutive SRC activation due to autocrine PDGF/PDGFR stimulation is observed in genetically-engineered mice that spontaneously develop PDAC, and accelerates tumor development and metastasis [85]. Co-expression of PDGF and SRC is also associated with increased pro-tumorigenic WNT/β-catenin signaling, elevated serum PDGF, and a poorer survival in human PDAC patients [85]. In another study, treatment of mice with the dual PDGFR and SRC tyrosine kinase inhibitor GN963 significantly reduced the growth of orthotopic L3.6pl tumors, and synergized with gemcitabine to abrogate liver metastasis [86].

### Cancer cell stemness

Increased SRC activity is observed in pancreatic cancer stem cells [87], and is linked to ligand/receptor signaling pathways commonly implicated in tumor progression [88–90]. Accordingly, therapeutic inhibition of SRC reduces pancreatic cancer stem cell abundance, as well as their colony forming and self-renewing properties in vivo [87].

### Hypoxia and metabolic reprograming

In response to hypoxia, pancreatic cancer cells adopt an invasive and treatment-resistant phenotype that enhances tumor growth and metastasis [91–94]. Hypoxia is also accompanied by an accumulation of immunosuppressive myeloid cells and cancer-associated fibroblasts, which promote the exhaustion and exclusion of cytotoxic effector cells [95–97].

SRC activity is increased in hypoxic regions of PDAC tumor-bearing mice, and can be therapeutically targeted using the small molecule SRC inhibitor AZD0530 [98]. Hypoxia-induced activation of SRC also stimulates downstream activation of FAK, NFκB, HIF1α, and STAT3 in cancer cells, which enhances survival, invasion, metastasis, and chemoresistance [99–102].

Tumor cells alter their metabolic needs in response to hypoxia to maintain their survival and proliferation [103]. This process, termed the Warburg effect, describes a process by which cancer cells preferentially use glycolysis for energy production [104]. The Warburg effect is advantageous as it provides rapidly proliferating tumor cells with metabolic intermediates to synthesize cellular components, improves the metastatic potential of cancer cells, and limits oxidative stress [105–108]. SRC has been shown to drive the Warburg effect and therapy resistance by inactivating pyruvate dehydrogenase (PDH), which regulates the metabolic fine-tuning between glycolysis and oxidative phosphorylation [109]. Conversely, treatment of cancer cells with the small molecule SRC inhibitors SU6656 and saracatinib increases PDH phosphorylation and the generation of reactive oxygen species [109]. Together, these findings demonstrate a key involvement of SRC via induction of molecular pathways involved in hypoxia and metabolic reprograming.

### Cancer cell invasion and metastasis

SRC expression is most prominent at the invasive border of PDAC, and correlates with enhanced epithelial to mesenchymal transition (EMT) and metastasis [110]. Loss of E-cadherin is a hallmark of EMT [111], and is associated with increased tumor cell invasion and spread [112, 113]. SRC is inversely correlated with E-cadherin levels in human PDAC, while its inhibition restores E-cadherin expression and decreases cellular invasion [114, 115]. The SRC/FAK signaling axis also promotes TGFβ-induced delocalization of E-cadherin [116], while concurrent inhibition of SRC and FAK prevents E-cadherin endocytosis and strengthens E-cadherin-mediated cell adhesions [117].

Detachment of normal cells from the extracellular matrix leads to reduced proliferation and cell death [118]. However, PDAC cells can evade this process by activating signaling pathways that enable adhesion-independent survival. Increased SRC auto-phosphorylation is observed following detachment of pancreatic cancer cells from the extracellular substratum, and results in the activation of downstream signaling proteins (e.g., AKT, JNK) important for cell survival and proliferation [119]. These findings reveal an additional mechanism by which aberrant activation of SRC facilitates tumor invasion and metastasis by preventing the death of migrating cancer cells.

Another way in which aberrant SRC activation contributes to invasion and metastasis is by facilitating the formation of invadopodia. Invadopodia are cell protrusions that mediate actin contractility, membrane trafficking, and focal degradation, thereby enhancing cancer cell extravasation during metastasis [120]. SRC and FAK signaling correlates with the matrix-remodeling ability of invadopodia [121, 122] by regulating the production of MMPs [123–125].

### Therapy resistance and modulating treatment response

Increased SRC expression positively correlates with chemoresistance in human pancreatic cancer cell lines [126], while oncogenic KRAS mutations induce a SRC-dependent amplification loop that promotes metastasis and therapy resistance in human PDAC tumors [127]. Conversely, therapeutic or siRNA-mediated SRC inhibition restores sensitivity to gemcitabine [126], 5-flourouracil [128], paclitaxel [129], and cetuximab [130] in preclinical PDAC models. Although the mechanisms underpinning a role for SRC in PDAC chemo-resistance are still unclear, these findings support the use of SRC inhibitors as a complementary strategy to improve response of pancreatic tumors to chemotherapy.

## Effects of tumor cell-extrinsic SRC activity in pancreatic cancer

Aberrant activation of SRC in cells of the immediate tumor environment can also promote PDAC development and progression via several mechanisms, including inflammation, immune modulation, desmoplasia, angiogenesis, and lymph-angiogenesis (Fig. 3).

### Inflammation and immune modulation

Chronic inflammation is a key component of PDAC, and is linked to tumor progression, metastasis, and chemoresistance [131]. In response to chronic inflammation, pancreatic acinar cells de-differentiate into a ductal-like phenotype in a process known as acinar-to-ductal metaplasia (ADM) [132]. ADM lesions may further develop into pancreatic intraepithelial neoplasia (PanIN) [132], which represent the dominant precursor to PDAC [133, 134].

Increased SRC expression is observed during the progression of chronic pancreatitis to PanIN and PDAC [135], while treatment of mice with the SRC kinase inhibitor PP2 significantly reduces the severity of caerulein-induced pancreatitis in mice and is associated with impaired activation of inflammatory signaling pathways (e.g., STAT3, ERK, NFκB) [136]. Mechanistically, aberrant activation of SRC is observed in circulating monocytes and tissue macrophages during chronic pancreatitis, as well as in tumor-associated macrophages and acinar cells [28, 137]. Accordingly, SRC regulates the production of IL6 by inflammatory macrophages [138], which is required for ADM and progression to PDAC [139]. SRC is also downstream of the SDF1/CXCR4 signaling axis [140], which facilitates mobilization of inflammatory leukocytes and bone marrow-derived mesenchymal cells during pancreatitis and tumor development [141, 142], and enables pancreatic cancer cell invasion and EMT [143, 144].

In addition to inflamed tissues, macrophages are also a major component of PDAC tumors and are associated with poor patient survival [96, 145]. SRC expression and activation is increased in PDAC-associated macrophages compared with resident macrophages in the normal pancreas, and correlates with tumor growth [137]. Activation of SRC in tumor-associated macrophages is induced by tumor-derived cytokines and chemokines (e.g., TNF, MIP, SDF-1), which amplify the production of inflammatory cytokines (e.g., TNF, IL1β, IL6) to reciprocally activate SRC in a feed-forward loop and promote PDAC progression [28, 146].

Surprisingly, the immune modulatory roles of SRC in PDAC remains understudied compared to other cancer types. In preclinical models of B16.OVA melanoma, 1956 sarcoma, MC38 colon, and 4T1 breast cancer, therapeutic inhibition of SRC was shown to enhance antitumor immunity by increasing the infiltration of T- and NK cells, and by reducing the abundance of regulatory T-cells (Tregs) [147]. Similar findings were observed in a mouse model of head and neck squamous cell carcinoma, where dasatinib dramatically reduced tumor growth by inhibiting the recruitment of myeloid-derived suppressor cells [148].

### Desmoplasia

Cancer-associated fibroblasts play a major role in the desmoplastic reaction of PDAC via extracellular matrix deposition and remodeling, production of growth factors, as well as reciprocal signaling interactions with cancer and immune cells to promote an immune-suppressive tumor microenvironment [149]. Conversion of normal fibroblasts into cancer-associated fibroblasts is modulated by YAP1-mediated activation of SRC, which stimulates cytoskeletal protein activation and actomyosin contractility [150].

Crosstalk between cancer-associated fibroblasts and the extracellular matrix reinforces the stiffness of the tumor stroma [151]. For example, binding of membrane-bound integrin receptors to extracellular matrix proteins triggers downstream activation of FAK and SRC to induce cytoskeletal remodeling and reinforce cellular stiffness [152]. Reciprocally, increased extracellular matrix stiffness activates the SRC/YAP/MYL9/MYL2 axis in cancer-associated fibroblasts to maintain their tumorigenic phenotype [153]. In contrast, concurrent inhibition of SRCand EGFR increases microvessel density and prevents fibrosis in orthotopic and genetically-engineered PDAC mouse models [154].

### Angiogenesis and lymph-angiogenesis

Angiogenesis plays a critical role in PDAC by providing oxygen and nutrients to cancer cells, facilitating tumor cell migration, and promoting the secretion of cytokines by endothelial cells to stimulate tumor growth [155]. VEGF is a key angiogenic molecule that is most frequently upregulated in tumor and immune cells [156], and several studies have demonstrated a requirement of SRC in VEGF-mediated angiogenesis by preventing endothelial cell apoptosis and influencing the stability of sprouting blood vessels [157–160].

Upregulated expression of IL8 is observed in PDAC, and enables tumor growth and metastasis by enhancing angiogenesis via paracrine interactions with endothelial cells [161, 162]. Of note, SRC activity correlates with IL8 production in human L3.6pl and PANC-1 pancreatic cancer cell lines, while pharmacologic inhibition of endogenous SRC or siRNA-mediated knock-down of SRC significantly reduces IL8 production and angiogenesis [56].

SRC also contributes to lymph-angiogenesis, which promotes metastasis to regional lymph nodes [163, 164]. The VEGF-C/VEGFR-3 signaling axis directly activates lymphatic endothelial cells and enhances the secretion of cytokines and growth factors that promote lymph vessel formation [165–168]. SRC promotes IL6-induced VEGF-C expression in lymphatic endothelial cells [169], and VEGF-C stimulation of lymphatic endothelial cells upregulates SRC activity [170]. This functional cooperation suggests paracrine interactions between SRC and VEGF-C signaling in the tumor microenvironment, since therapeutic inhibition of SRC suppresses VEGF-C expression in pancreatic cancer cells, and impairs the proliferation and sprouting of lymphatic endothelial cells [170].

## Targeting SRC in pancreatic cancer

**Fig. 4:**
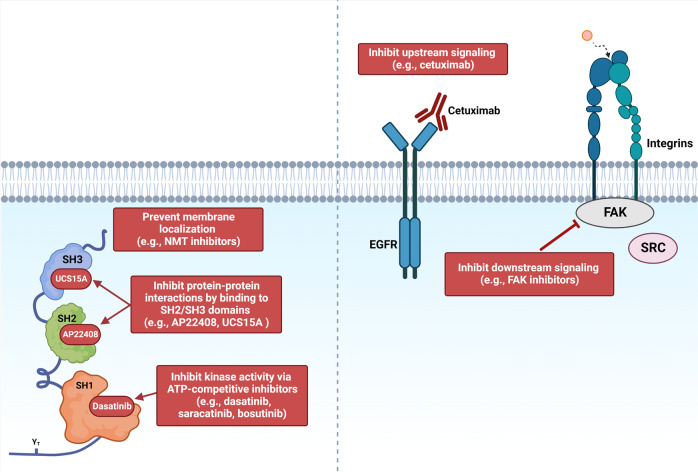
Therapeutic strategies to inhibit SRC signaling. Therapeutic approaches to inhibit SRC include (1) interfering with protein stability/membrane localization, (2) reducing protein-protein interactions, (3) inhibiting catalytic kinase activity, or (4) by interfering with upstream/downstream signaling components. *Figure created in Biorender*.

Strategies to inhibit SRC include suppressing its catalytic activity, inhibiting protein stability, interfering with signaling components of the SRC signaling pathway, or by reducing its protein-protein interactions (Fig. 4).

### Inhibiting SRC catalytic activity

Small molecule ATP-competitive SRC inhibitors that target the catalytic activity of SRC include dasatinib (Sprycel, BMS354825, Bristol-Myers Squibb), bosutinib (SKI-606, Wyeth), and saracatinib (AZD0530, AstraZeneca) [171]. The antitumorigenic activity of dasatinib has been extensively studied in preclinical models; however, its efficacy in Phase I/II clinical trials has been disappointing (Table 1). One major issue is the lack of biomarkers to identify patients who are most likely to respond to therapy. This is crucial, since the therapeutic efficacy of SRC inhibitors is influenced by the level of SRC activity in tumors [135]. In one trial, dasatinib failed to improve the overall survival of patients with metastatic pancreatic cancer; however, a durable response was observed in a few patients following brief exposure to therapy (NCT00474812) [172]. These findings provide compelling rationale for studying the biology of “exceptional responders” to identify biomarkers or genetic vulnerabilities that could be exploited to improve treatment response [173]. Currently, small molecule SRC inhibitors are not FDA-approved for the treatment of solid tumors.

**Table 1:** Summary of selected clinical trials using SRC inhibitors in pancreatic cancer.

Drug	Selection criteria	Phase	Combination partners	Side effects	Status	Outcome	References
Dasatinib	Locally-advanced pancreatic cancer	II	Gemcitabine	Neutropenia, fatigue, thrombocytopenia, anemia, abdominal pain	Complete	No significant clinical activity	[226]
	Advanced pancreatic cancer	I	Erlotinib + gemcitabine	Anemia, fatigue, nausea, lymphopenia, leukopenia, neutropenia, thrombocytopenia	Active, not recruiting	Well tolerated. Stable disease as best response observed in 69% of patients	NCT01660971 [201]
	Metastatic pancreatic cancer	II	Monotherapy	Terminated due to toxicity	Terminated due to toxicity	N/A	NCT00544908
		II	Monotherapy	Fatigue, nausea, edema, pleural effusions	Completed, awaiting results	No significant clinical activity	NCT00474812 [172]
		II	5-FU and oxaliplatin	Nausea, fatigue, neutropenia, upper gastrointestinal hemorrhage, depression	Complete	Well tolerated. No improvement in efficacy over chemotherapy alone	NCT01652976 [227]
		II	mFOLFOX6	Results pending	Completed	Results pending	NCT01652976
	Resected pancreatic cancer (adjuvant)	II	Gemcitabine	Results pending	Completed	Results pending	NCT01234935
Bosutinib	Advanced solid cancers (including pancreatic)	I	Monotherapy	Nausea, diarrhea, vomiting, fatigue, anorexia	Completed	Well tolerated. Complete response and stable disease observed	NCT00195260 [228]
	Resected pancreatic cancer	I	Gemcitabine	N/A	Terminated due to slow accrual	N/A	NCT01025570
	Locally advanced/metastatic solid cancers (incl pancreatic)	I/II	Capecitabine	Diarrhea, nausea, vomiting	Terminated (reason unknown)	Limited efficacy	NCT00959946 [229]
Saracatinib	Advanced pancreatic cancer	I/II	Gemcitabine	Anorexia, diarrhea, anemia, fatigue,	Completed	Well tolerated. No improvement in efficacy over chemotherapy alone	NCT00265876 [230]
	Recurrent metastatic pancreatic cancer	II	Monotherapy	Results pending	Completed	Results pending	NCT00735917

### Interfering with protein stability

Another approach to reduce SRC activation includes triggering protein instability or preventing maturation of the protein. The molecular chaperone Heat shock protein 90 (Hsp90) promotes the stability and function of oncoproteins including SRC [174]. Hsp90 inhibitors are divided into several classes based on their mechanism of action, including (1) inhibiting ATP binding, (2) disruption of co-chaperone/Hsp90 interactions, (3) preventing post-translational modifications of Hsp90, and (4) interfering with client/Hsp90 associations [174]. The latter class of Hsp90 inhibitors promote the degradation of client proteins [175], and have shown promising results in Phase I clinical trials [174–176]. Given that Hsp90 is required for the maturation of SRC [177], Hsp90 inhibitors may represent an effective strategy for the treatment of PDAC. Indeed, Hsp90 inhibitors ICPD47 and ICPD62 have been shown to synergize with chemotherapy and reduce the growth of pancreatic cancer cell lines in vitro [178]. Furthermore, treatment of mice with the Hsp90 inhibitor XL888 in combination with anti-PD1 impaired the growth of subcutaneous Panc02 and orthotopic KPC tumors [179]. Further analysis revealed that tumors of mice treated with both XL888 and anti-PD1 showed increased T-cell infiltration and an enrichment of genes associated with immune activation [179]. In separate studies, inhibition of Hsp90 also sensitized treatment-refractory PDAC xenografts to chemotherapy and radiotherapy [180]. A Phase I/II trial of XL888 in combination with anti-PD1 is currently undergoing clinical evaluation in patients with advanced pancreatic cancer (NCT3095781).

N-myristoyltransferases (NMTs) are enzymes that regulate the function of oncogenic proteins by catalyzing myristoylation [181]. Protein N-myristoylation of SRC anchors it to the cell membrane, and helps maintain its structure and kinase activity [181, 182]. Interestingly, NMT levels correlate with SRC activation in human tumors, and is associated with a poor prognosis [183]. Meanwhile, inhibition of NMT1 suppresses SRC-induced oncogenic signaling and significantly reduces the growth of tumor xenografts with limited toxicity in vivo [183]. Loss of myristoylation also suppresses downstream SRC signaling pathways, including FAK and MAPK [184].

### Interfering with upstream and/or downstream molecules of the SRC signaling pathway

Given the complexity of the SRC signaling network, therapeutic agents aimed at interfering with upstream and/or downstream SRC signaling components (e.g., integrins, EGFR, FAK, PI3K) represent another promising approach for the treatment of PDAC (Table 2).

**Table 2:** Summary of selected clinical trials targeting upstream and/or downstream activators of SRC in pancreatic cancer.

Target	Drug	Selection criteria	Phase	Combination partners	Side effects	Status	Outcome	References
Integrins	Cilengitide	Advanced pancreatic cancer	II	Gemcitabine	Nausea, dyspepsia, dyspnea, chills, fever	Completed	Well tolerated; no significant improvement in survival	EMD 121974 [190]
	IMGN388	Advanced solid cancers (including pancreatic cancer)	I	Monotherapy	Results pending	Completed	Results pending	NCT00721669 [231]
	Volociximab	Metastatic pancreatic cancer	II	Gemcitabine	Results pending	Completed	Results pending	NCT00401570 [232]
EGFR	Erlotinib	Locally advanced pancreatic cancer	III	Gemcitabine	Anemia, neutropenia, diarrhea, rash	Completed	No significant improvement in overall survival	NCT00634725 [193]
		Advanced pancreatic cancer	II	Gemcitabine	Neutropenia, lymphopenia, fatigue	Completed	Well tolerated; no significant improvement in survival	NCT00810719 [233]
		Advanced pancreatic cancer	III	Gemcitabine	Nausea, vomiting, fatigue, anorexia	Completed	Increased overall and progression free survival in combination group	NCT00026338 [192]
	Cetuximab	Advanced pancreatic cancer	III	Gemcitabine	Diarrhea, rash, fatigue, nausea, vomiting	Completed	No significant improvement in overall survival	NCT00075686 [234]
	Nimotuzumab	Advanced pancreatic cancer	II	Gemcitabine	Fatigue, rash	Completed	Well tolerated. Significantly improved progression free and overall survival	NCT00561990 [235]
FAK	PF‐00562271	Advanced solid cancers (including pancreatic cancer)	I	Monotherapy	Headache, nausea/vomiting, dehydration, edema	Completed	MTD determined	NCT00666926 [236]
	VS‐4718	Advanced pancreatic cancer	I	Gemcitabine/ Nab‐paclitaxel	N/A	Terminated	N/A	NCT02651727
	Defactinib	Advanced solid cancers (including pancreatic cancer)	II	Pembrolizumab (anti‐PD1)	N/A	Recruiting	N/A	NCT02758587
		Advanced solid cancers (including pancreatic cancer)	II	Pembrolizumab and Gemcitabine	Fatigue, nausea, myalgia, nausea/vomiting, anorexia, fever	Completed	Well tolerated. No partial or complete responses observed	NCT02546531
	GSK2256098	Recurrent pancreatic cancer	II	Trametinib (MEK1/2 inhibitor)	N/A	Active, not recruiting	N/A	NCT02428270
PI3K/AKT	Oleandrin (PBI‐05204)	Metastatic pancreatic cancer	I	Monotherapy	N/A	Active, not recruiting	N/A	NCT02329717
	AZD5363	Advanced/recurrent solid cancers (including pancreatic cancer)	II	Monotherapy	N/A	Recruiting	N/A	NCT02465060
	Perifosine	Advanced pancreatic cancer	II	Monotherapy	Nausea, vomiting, fatigue	Completed	No objective response observed	NCT00059982 [237]
	Buparlisib	Advanced solid cancers (including pancreatic cancer)	I	mFOLFOX6	Neutropenia, fatigue, leukopenia, hyperglycemia, thrombocytopenia	Completed	MTD determined	NCT01571024 [238]
		Advanced solid cancers (including pancreatic cancer)	I	Trametinib (MEK1/2 inhibitor)	Stomatitis, diarrhea, dysphagia, rash	Completed	Minimal activity observed in pancreatic cancer patients	NCT01155453
	MK2206	Advanced pancreatic cancer	I/Ib	Dinaciclib (CDK inhibitor)	Results pending	Completed	Results pending	NCT01783171
		Recurrent metastatic pancreatic cancer	II	Selumetinib (MEK1/2 inhibitor)	Nausea, vomiting, fatigue, anorexia	Completed	No improvement in overall survival.	NCT01658943 [239]
	Afuresertib (GSK2110183)	Advanced solid cancers (including pancreatic cancer)	I/II	Trametinib (MEK1/2 inhibitor)	Diarrhea, dermatitis, rash, fatigue, nausea, vomiting	Completed	Poor tolerability: no patients enrolled in Phase II	NCT01476137 [240]
	Uprosertib (GSK2141795)	Advanced solid cancers (including pancreatic cancer)	I	Trametinib (MEK1/2 inhibitor)	Diarrhea, rash	Completed	Poor tolerability and minimal clinical activity	NCT01138085 [241]

*MTD* Maximum tolerated dose.

#### Integrins

Integrins are transmembrane receptors that bind with proteins (e.g., vinculin, filamin) to regulate cytoskeleton stability, and phosphorylate kinases (e.g., SRC, FAK) to activate downstream signaling pathways. Activation of SRC by β1 integrin enhances the invasive capacity of pancreatic cancer cells [185], while activation of SRC by β3 integrin promotes anchorage-independent PDAC tumor growth and lymph node metastasis [186]. Integrins are also involved in stellate cell activation [187], and the production of tumor-promoting cytokines [188]. In orthotopic and genetically-engineered models of PDAC, coadministration of cilengitide (avβ3 and avβ5 integrin antagonist) and verapamil (calcium channel blocker) increased chemo-sensitivity to gemcitabine, and significantly reduced tumor growth compared with monotherapy-treated groups [189]. However, a Phase II trial combining cilengitide with gemcitabine in patients with advanced pancreatic cancer did not achieve clinical benefit (EMD 121974) [190] (Table 2).

#### EGFR

Reciprocal signaling between SRC and EGFR contributes to a more cancer-aggressive phenotype by enhancing tumor cell proliferation, invasion, and metastasis [71, 78]. Two main approaches used to target EGFR include monoclonal antibodies (e.g., cetuximab, nimotuzumab) directed against the extracellular domain, as well as small molecule inhibitors (e.g., erlotinib) that compete for the ATP binding site in the tyrosine kinase domain [191]. Erlotinib is the furthest in development for the treatment of PDAC, but has demonstrated mixed results in the clinic (Table 2). In a Phase III trial, erlotinib in combination with gemcitabine significantly improved progression-free and overall survival (NCT00026338) [192], but failed to produce clinical activity in other studies (NCT00634725 [193], NCT00026338 [192]).

#### FAK

The SRC/FAK signaling axis is implicated in PDAC by increasing tumor cell proliferation, EMT, and metastasis [68, 69, 116, 117]. The FAK inhibitor SK2256098 attenuates the proliferation, motility, and survival of pancreatic cancer cells in vitro [194], while VS-4718 doubled the survival of tumor-bearing mice by restoring sensitivity to chemotherapy and immunotherapy [30, 195]. These changes were associated with reduced tumor fibrosis and decreased numbers of immunosuppressive cells [30]. Based on these encouraging findings, several trials combining FAK inhibitors with chemotherapy (e.g., gemcitabine, nab‐paclitaxel) or immunotherapy (e.g., anti-PD1) are currently underway (Table 2).

#### PI3K

SRC-mediated activation of PI3K [45, 46] results in downstream phosphorylation of AKT and enhances the growth and survival of pancreatic cancer cells [47]. Accordingly, treatment of mice with the pan-PI3K inhibitor LY294002 inhibited the growth of orthotopic PDAC tumors and decreased peritoneal and liver metastasis [196]. Several pan-PI3K inhibitors have been evaluated in Phase I/II clinical trials in patients with advanced PDAC (Table 2), but have shown poor tolerability and negligible clinical benefit.

### Reducing protein-protein interactions

Small molecule non-peptide inhibitors (e.g., AP22408, AP22161, UCS15A) have shown efficacy at reducing SRC protein-protein interactions; however, their effectiveness in cancer remains to be evaluated. AP22408 and AP22161 selectively bind to the SH2 domain of SRC [197, 198], while UCS15A prevents SH3 domain protein-protein interactions [199, 200].

## Rationale for combination therapy

Despite encouraging results in preclinical studies, SRC inhibitors have not produced significant clinical benefit in PDAC (Table 1). The development of innovative and rational drug combinations that incorporate SRC inhibition as an adjuvant therapy therefore represents a potential approach to improve patient outcomes with manageable side effects. However, additional studies are required to identify which combination partners are likely to produce the most clinical benefit.

### Combining SRC inhibitors with chemotherapy

Aberrant SRC activation plays a role in mediating chemoresistance in PDAC, while therapeutic inhibition of SRC restores chemo-sensitivity of human pancreatic cancer cells [128–130]. Likewise, concomitant inhibition of SRC and EGFR in combination with gemcitabine overcomes STAT3-mediated chemoresistance and attenuates the growth of PDAC xenografts [79]. Encouraging clinical activity was observed in a Phase I trial of patients with metastatic or locally-advanced PDAC, where dual SRC and EGFR inhibition (dasatinib plus erlotinib) in combination with gemcitabine resulted in stable disease in 69% of patients (NCT01660971) [201]. However, other Phase I/II trials combining dasatinib, bosutinib, or saracatinib with gemcitabine have failed to produce significant clinical activity (Table 1).

### Combining SRC inhibitors with targeted therapies

Given the synergism between multiple signaling pathways in PDAC, combining SRC inhibitors with additional targeted therapies represents another promising approach to induce robust antitumor responses. For example, inhibition of the SRC/EGFR axis in combination with gemcitabine dramatically reduced cancer cell proliferation, survival, and the growth of orthotopic tumors [79, 154, 201]. This triple combination was also shown to overcome STAT3-mediated chemoresistance and attenuate the growth of PDAC xenografts [79]. Likewise, SKLB261 (multikinase inhibitor of EGFR, SRC, and VEGFR2) potently suppressed the proliferation and invasion of human PDAC cells, restored chemo-sensitivity, and extended the survival of tumor-bearing mice [202]. Dual targeting of SRC and SHP2 (required for full activation of the RAS/ERK pathway) potently inhibited the growth of orthotopic PDAC tumors [203], while concurrent inhibition of STAT3, SRC, and EGFR increased gemcitabine chemo-sensitivity and significantly reduced the growth of PDAC xenografts [204]. Thus, multitargeted therapies have the potential to be more effective at inducing robust antitumor effects in PDAC than blockade of individual pathways alone.

### Combining SRC inhibitors with immunotherapy

The poor response of PDAC tumors to immunotherapy is largely attributed to a desmoplastic tumor microenvironment that is densely populated by cancer-associated fibroblasts and immunosuppressive myeloid cells, which promote the exhaustion and exclusion of cytotoxic effector cells [8]. Conversely, therapeutic inhibition of SRC reduces the growth of various solid tumors and hematological malignancies by enhancing the activation, proliferation, and recruitment of cytotoxic CD8 T cells and NK cells, suppressing the recruitment of myeloid-derived suppressor cells and Tregs, and by inhibiting the tumorigenic phenotype of cancer-associated fibroblasts [147, 148, 150, 151]. Thus, SRC inhibition may represent a promising adjunct to immunotherapy; however, the therapeutic benefit of combining SRC inhibitors with immune checkpoint blockade has not been extensively studied in the context of PDAC.

## Clinical challenges and therapeutic perspectives

Contributing factors that underpin the poor response to SRC inhibitors in the clinic include the highly aggressive nature of PDAC, rapid development of drug resistance, and lack of patient stratification to identify those who are most likely to benefit from treatment. To maximize the therapeutic benefit from incorporating SRC inhibition into existing cancer treatments, several challenges need to be addressed. One major issue is the lack of effective biomarkers to identify patients who are most likely to benefit from SRC inhibitors. This is crucial, since the therapeutic efficacy of SRC inhibitors is influenced by the extent of SRC activity in tumors [135, 205–207], as well as mutations in other signaling proteins (e.g., c-MET and STAT3) [135].

### Maximizing clinical translation

To date, all completed clinical trials of SRC inhibitors in pancreatic cancer have been performed in unselected patients that failed standard-of-care therapies. Genomic analyses have identified the existence of genetically-distinct PDAC subtypes, including: (1) squamous, (2) pancreatic progenitor, (3) immunogenic, and (4) aberrantly differentiated endocrine exocrine [42]. In one study, synergism between SRC inhibitors (e.g., dasatinib, PP2) and a MEK1/2 inhibitor (pimasertinib) enhanced sensitivity to gemcitabine in the squamous subtype of pancreatic cancer cells (e.g., SW1990 and BxP3) and not in PDAC progenitor cells (e.g., AsPC1) [208]. Likewise, clinical stage correlates with increased expression of phosphorylated SRC, and higher baseline levels of phosphorylated SRC is associated with improved progression-free survival following dasatinib therapy [135, 205–207]. Thus, assessment of SRC activation based on phosphorylation status or gene expression analysis may serve as a biomarker to stratify and identify patients who are most likely to benefit from therapy.

Although SRC inhibitors have failed to produce clinical benefit in most PDAC patients, durable and sustained responses are observed in a small subset of patients following brief exposure to therapy (NCT00474812) [172]. Further investigations into the biology of these exceptional responders are warranted to identify biomarkers and/or molecular characteristics that could be exploited to maximize therapeutic response. Specifically, patient-derived tumor organoids or xenografts may help reveal key mechanistic insights to guide the design of clinical trials [173].

Given the unequivocal role of SRC in promoting tumor cell invasion and migration, there is also a need to evaluate the efficacy of SRC inhibitors in early-stage disease or in the adjuvant setting after tumor resection. However, since most PDAC patients are diagnosed with metastasis, recruitment of patients at early stages of the disease remains a significant challenge.

Since the redundancy of cellular pathways may limit the efficacy of inhibiting SRC alone, multitargeted therapies that also block EGFR or STAT3 represent an effective strategy to boost antitumor responses. In line with these findings, encouraging clinical activity was observed in a Phase I trial of patients with metastatic or locally-advanced PDAC, where dual SRC and EGFR inhibition (dasatinib plus erlotinib) in combination with gemcitabine resulted in stable disease in 69% of patients (NCT01660971) [201]. In another study, STAT3 activation correlated with dasatinib resistance in pancreatic cancer cells [135], while dual inhibition of STAT3 and SRC resulted in significantly smaller PDAC tumors in mice compared to monotherapy-treated groups [204]. Likewise, MET amplification mediates resistance to SRC inhibitors in various solid malignancies, while concurrent inhibition of SRC and MET produces a synergistic cytotoxic effect on tumor growth [209–211]. Collectively, these findings provide compelling rationale for the design of innovative and rational combinatorial strategies to improve the clinical activity of SRC inhibitors.

### Clinical considerations for direct vs. indirect inhibition of SRC

Although second-generation SRC inhibitors (e.g., Bosutinib, Dasatinib, Saracatinib) are designed to be more selective and potent compared to first-generation inhibitors (e.g., PP1, SU6656), these multikinase inhibitors still confer off-target effects and toxicity in normal cells and tissues. For example, dasatinib inhibits the proliferation and activation of primary human T cells [212], impairs humoral immunity by promoting B-cell apoptosis [213], and reduces the pro-inflammatory capacity of human neutrophils [214].

Inhibition of SRC may also negatively impact bone homeostasis, since SRC plays an essential role in regulating osteoclastic bone resorption and osteoblastic bone formation. Accordingly, *Src* deficiency results in osteopetrosis in mice [215–218], while dasatinib treatment increases bone mass by reducing bone resorption and stimulating bone anabolism [219]. Thus, the SRC SH2 inhibitors AP22161 and AP22408, which preferentially accumulate on the surface of bones exert potent inhibition of osteoclast-mediated bone resorption [197].

Other studies suggest that SRC inhibition may perturb platelet activation and aggregation, since *Src-*deficient platelets demonstrate reduced spreading on fibrinogen, and dasatinib treatment increases the tail bleeding time of mice in a dose-dependent manner [220–222]. Likewise, the SRC inhibitors PP2, SU6656, and dasatinib potently inhibit the coagulation-promoting and clot-retracting activities of human platelets [221, 223]. These findings raise important clinical implications, since gastrointestinal, genitourinary, soft tissue hematoma, and central nervous system bleeding is observed in up to 40% of patients during dasatinib therapy [224]. This issue is further complicated by the observation that most patients with bleeding episodes in response to treatment with these drugs also exhibit low platelet counts and advanced-stage cancers that require higher doses of dasatinib for clinical benefit [225].

As an alternative to broad-spectrum SRC inhibitors, indirect strategies that interfere with upstream and/or downstream molecules within the SRC signaling cascade may represent safer and more selective approaches, since these molecules are often less ubiquitously expressed. Likewise, targeting downstream effectors may overcome resistance mechanisms that arise from compensatory activation of other SCR family kinases. Thus, additional studies comparing the advantages between indirect and direct SRC inhibition are warranted.

## Concluding remarks

Given the diverse roles of SRC in PDAC and association with a poor prognosis, SRC represents a promising therapeutic target for pancreatic cancer. However, since direct inhibition of SRC still suffers from sufficient target specificity and may have deleterious consequences on cellular processes, alternative approaches aimed at interfering with upstream and/or downstream molecules of the SRC signaling pathway may represent a safer option. Despite encouraging results in preclinical studies, SRC inhibitors have also failed to produce clinical benefit in most PDAC patients. Contributing factors that underpin the poor response to therapy include the highly aggressive nature of PDAC, complexity of the SRC signaling network, rapid development of drug resistance, and lack of patient stratification to identify those who are most likely to benefit from treatment. Thus, additional studies are needed to better understand the diverse roles of SRC in PDAC biology, and to identify prognostic and predictive factors to help stratify patients and maximize therapeutic response.

## Data Availability

Data sharing is not applicable to this article as no datasets were generated or analyzed during the current study.
